# Supplementary material for: Multi-omics profiling reveals rhythmic liver function shaped by meal timing
Source: Nat Commun. 2023 Sep 29;14:6086. doi: 10.1038/s41467-023-41759-9 (PMC10541894; doi:10.1038/s41467-023-41759-9)
Supplement: Supplementary file 1 — Supplementary Information [file 41467_2023_41759_MOESM1_ESM.pdf]

SUPPLEMENTARY INFORMATION

Multi-omics Profiling Reveals Rhythmic Liver Function Shaped by Meal Timing,
Huang, Chen, Zhou, Xin, et al.

Supplementary Figures

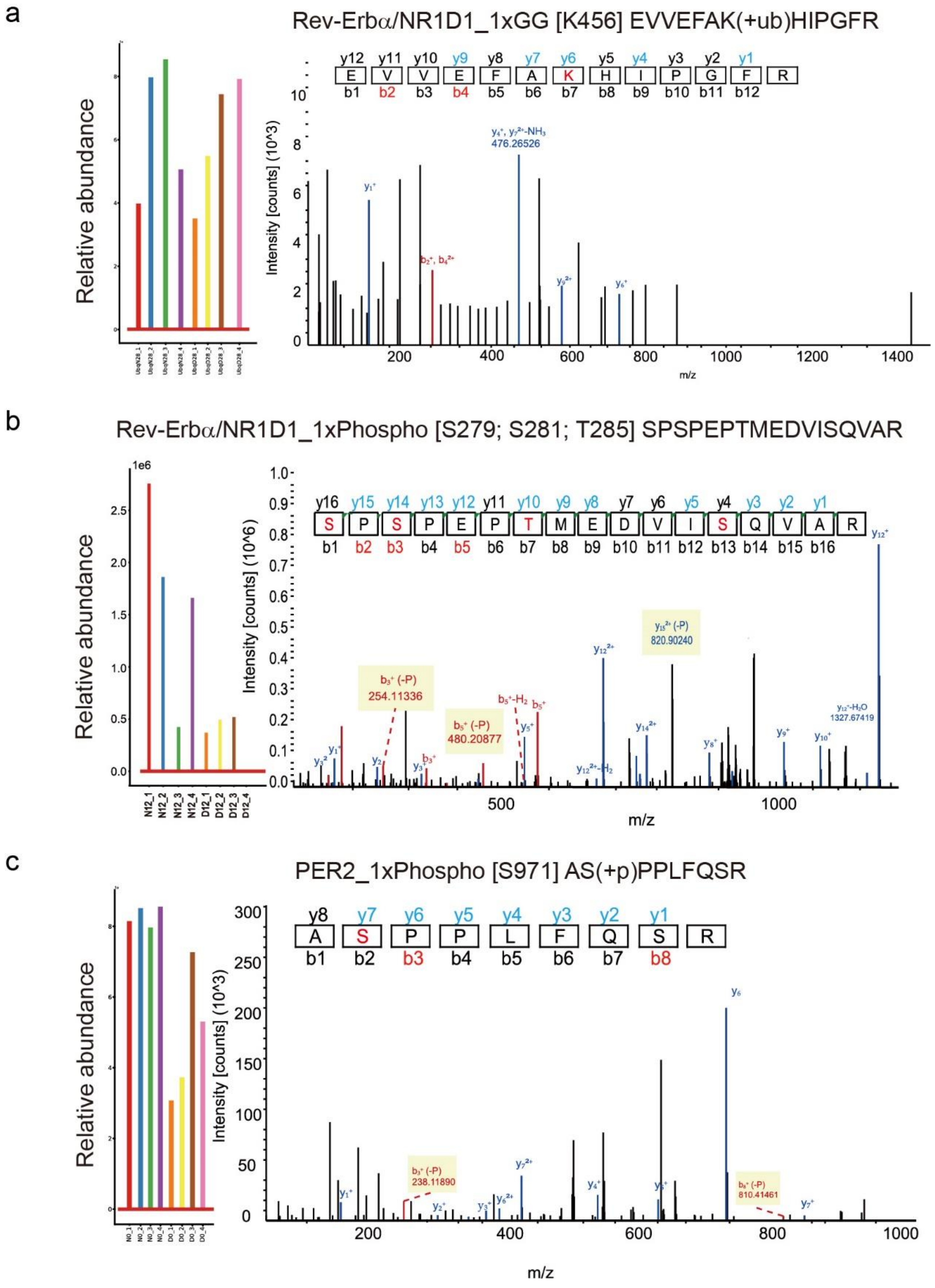

Supplementary Figure 1. Mass spectrometric signatures of modification sites on clock proteins. (a) Secondary mass spectra related to the residual ubiquityl-peptide derived from Rev-Erbα/NR1D1. (b) Secondary mass spectra related to a phospho-peptide derived from NR1D1. (c) Secondary mass spectra related to a phospho-peptide derived from PER2. Source data are provided as a Source Data file.

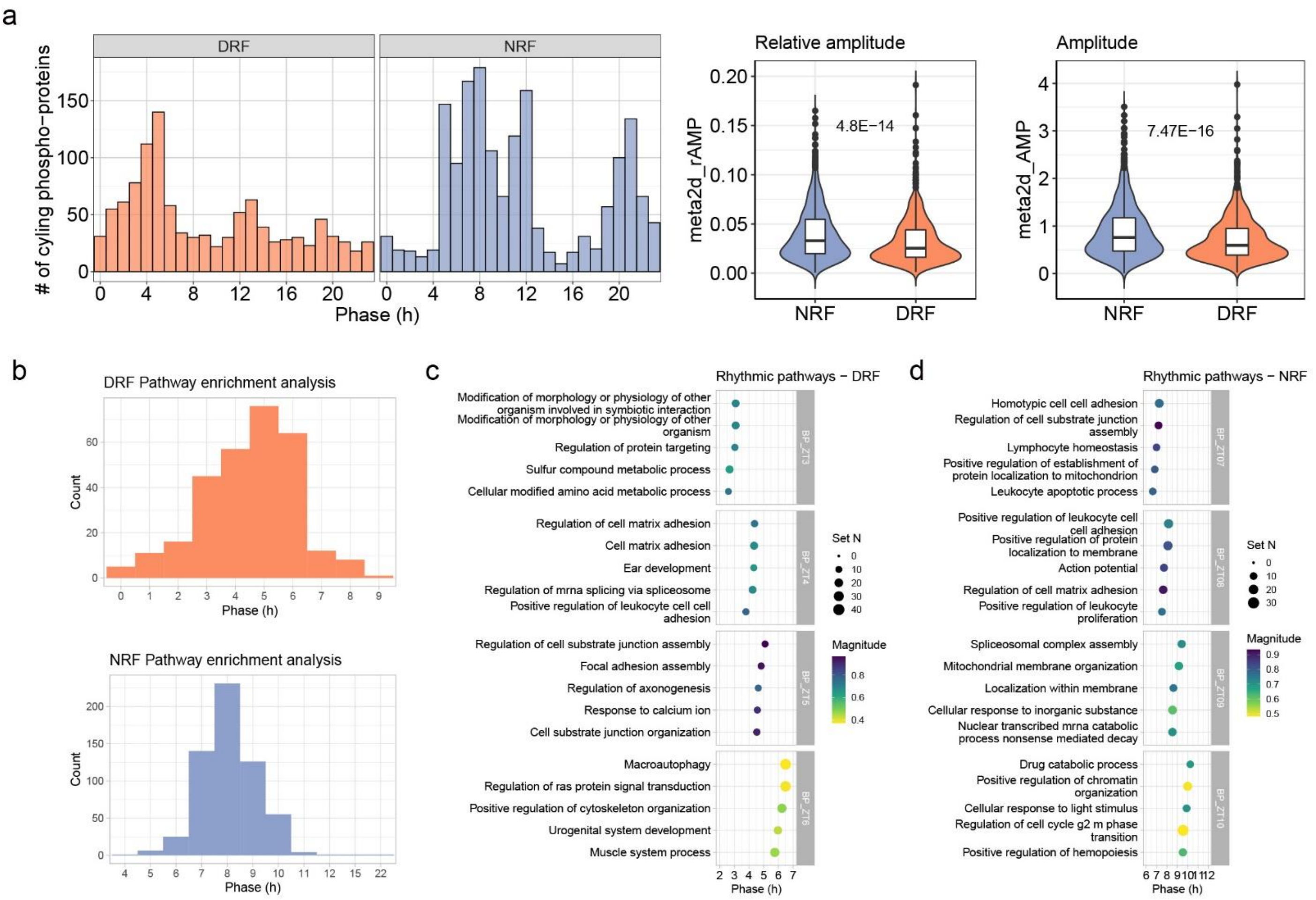

Supplementary Figure 2. Phase and pathway analysis of diurnal phospho-proteome in livers from TRF female mice. (a) Phase distribution and amplitude analysis of cycling phosphoproteins in livers from TRF female mice, the rhythmicity of which is defined by RAIN (adjusted $P < 0.05$), MetaCycle (adjusted $P < 0.05$) and Circacompare ($P < 0.05$) (n = 48 mice per group across 12 time points covering two diurnal cycles). Two-sided unpaired Wilcoxon test. (b) Histogram showing the phase distribution of enriched rhythmic pathways under DRF or NRF, as measured by PSEA of cycling phosphoproteins in livers from DRF or NRF female mice (Kuiper test, $q < 0.05$). (c-d) Representative rhythmic pathways in mouse livers under DRF (c) or NRF (d), as shown by the hour of their estimated peak time. Source data are provided as a Source Data file.

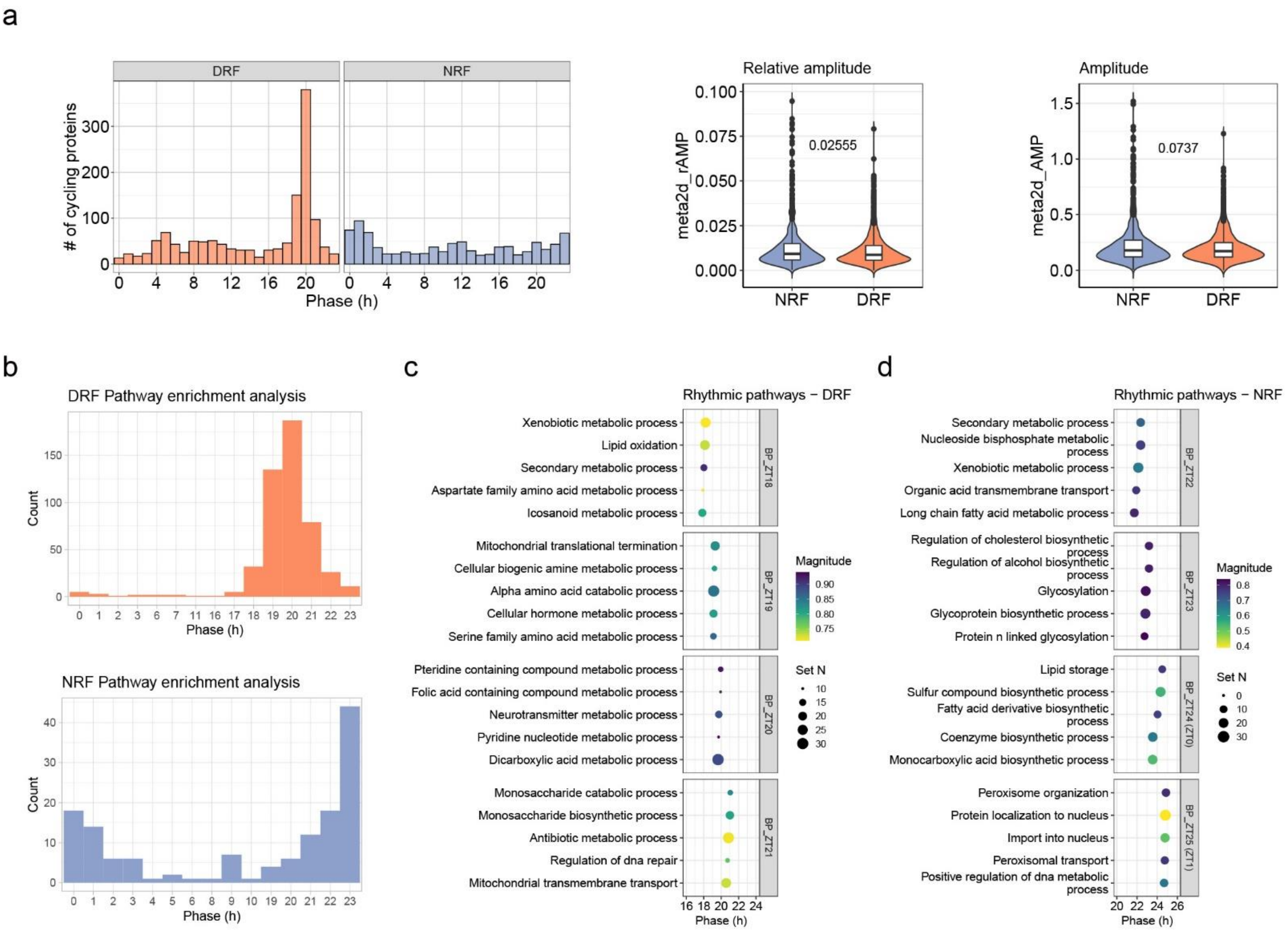

Supplementary Figure 3. Phase and pathway analysis of diurnal proteome in livers from TRF female mice. (a) Histograms showing phase distribution and amplitude of cycling proteins in livers from TRF female mice, the rhythmicity of which is defined by RAIN (adjusted $P < 0.05$), MetaCycle (adjusted $P < 0.05$) and Circacompare ($P < 0.05$) (n = 48 mice per group across 12 time points covering two diurnal cycles). Two-sided unpaired Wilcoxon test. (b) Histogram showing the phase distribution of enriched rhythmic pathways under DRF or NRF, as measured by PSEA of cycling proteins in livers from DRF or NRF female mice (Kuiper test, $q < 0.05$). (c-d) Representative rhythmic pathways in mouse livers under DRF (c) or NRF (d), as shown by the hour of their estimated peak time. Source data are provided as a Source Data file.

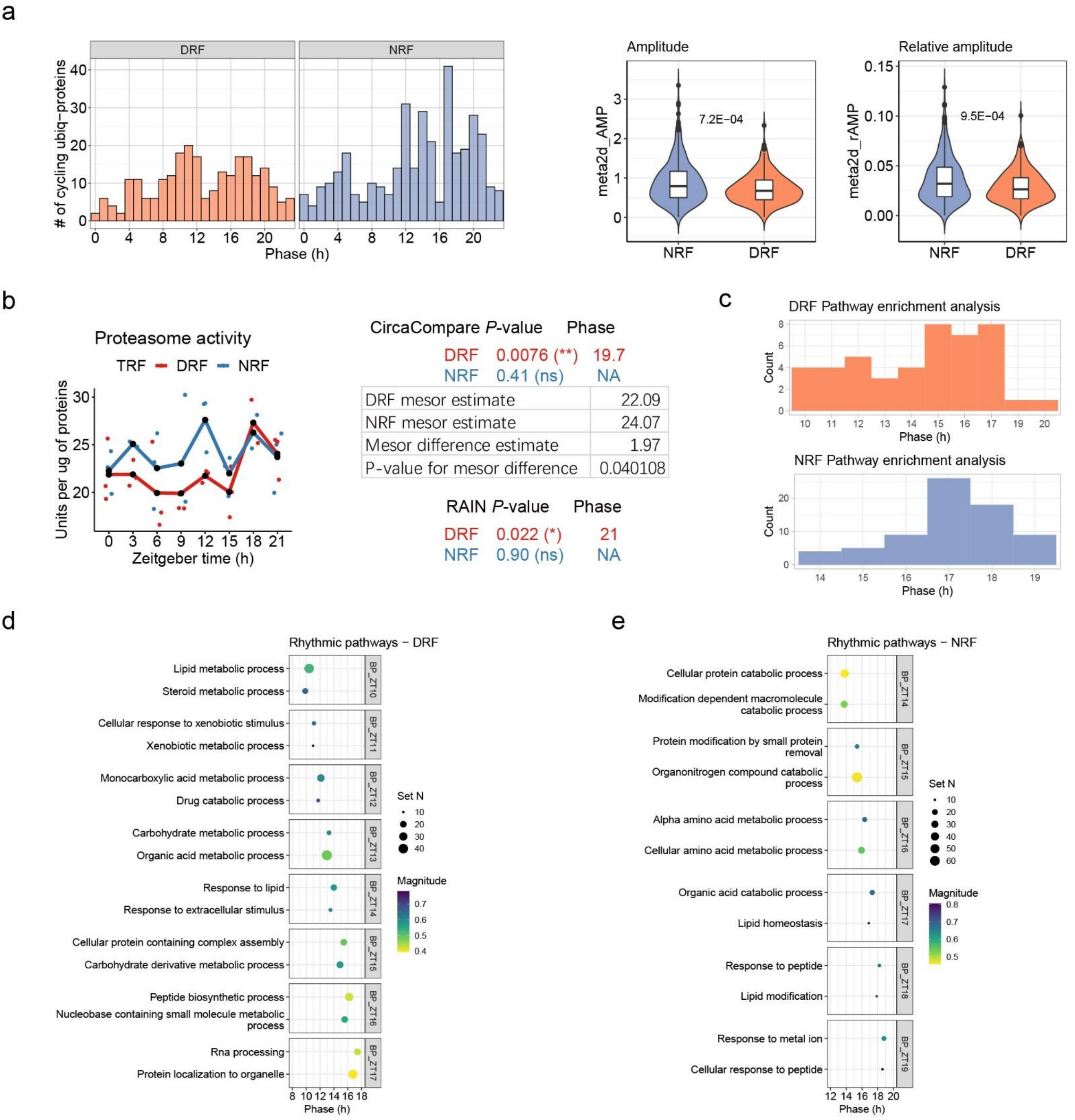

Supplementary Figure 4 Phase and pathway analysis of diurnal ubiquitylproteome in livers from TRF female mice. (a) Histograms showing phase distribution and amplitude of cycling ubiquityl-proteins in livers from TRF female mice, the rhythmicity of which is defined by RAIN (adjusted $P < 0.05$), MetaCycle (adjusted $P < 0.05$) and Circacompare ($P < 0.05$) ($n = 48$ mice per group across 12 time points covering two diurnal cycles). Two-sided unpaired Wilcoxon test. (b) Diurnal proteasome activity in livers from TRF female mice ($n = 24$ mice per group across 8 time points covering one diurnal cycle). Rhythmicity statistics were calculated by Circacompare and RAIN; ns, not significant;

*P < 0.05; **P < 0.01. (c) Histogram showing the phase distribution of enriched rhythmic pathways under DRF or NRF, as measured by PSEA of cycling proteins in livers from DRF or NRF female mice (Kuiper test, q < 0.05). (d-e) Representative rhythmic pathways in mouse livers under DRF (d) or NRF (e), as shown by the hour of their estimated peak time. Source data are provided as a Source Data file.

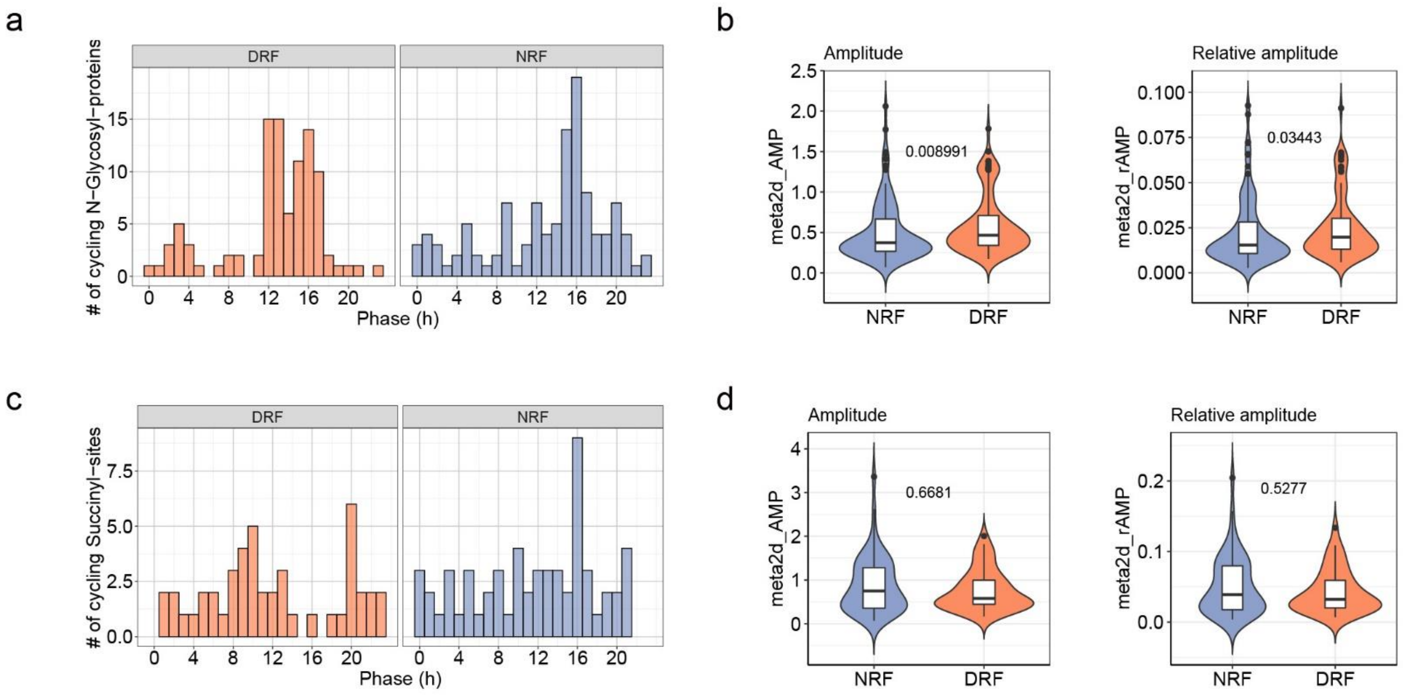

Supplementary Figure 5. Phase analysis of diurnal N-glycosylproteome and succinylome in livers from TRF female mice. (a-b) Histograms showing phase distribution and amplitude of cycling N-glycosyl proteins in livers from TRF female mice, the rhythmicity of which is defined by RAIN (adjusted P < 0.05), MetaCycle (adjusted P < 0.05) and Circacompare (P < 0.05) (n = 24 mice per group across 12 time points covering two diurnal cycles). Two-sided unpaired Wilcoxon test. (c-d) Histograms showing phase distribution and amplitude of cycling succinyl-sites among liver proteins from TRF female mice, the rhythmicity of which is defined by RAIN (adjusted P < 0.05), MetaCycle (adjusted P < 0.05) and Circacompare (P < 0.05) (n = 24 mice per group across 12 time points covering two diurnal cycles). Two-sided unpaired Wilcoxon test. Source data are provided as a Source Data file.

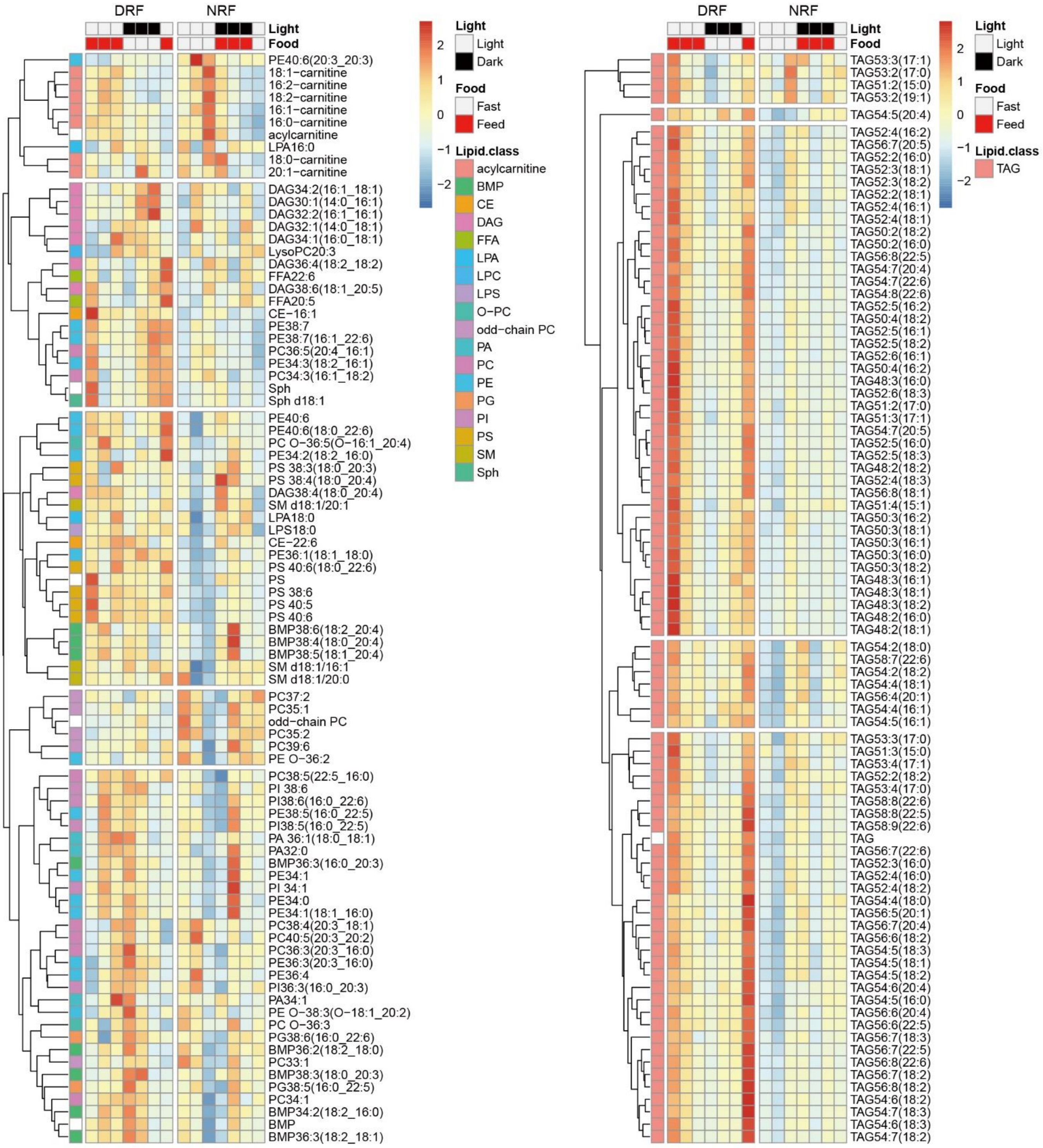

Supplementary Figure 6. Global profiling of lipids in livers from TRF mice. Diurnal levels of cycling hepatic lipids were shown in heatmap (*n* = 28 per group across 7 time points covering one diurnal cycle). Source data are provided as a Source Data file.

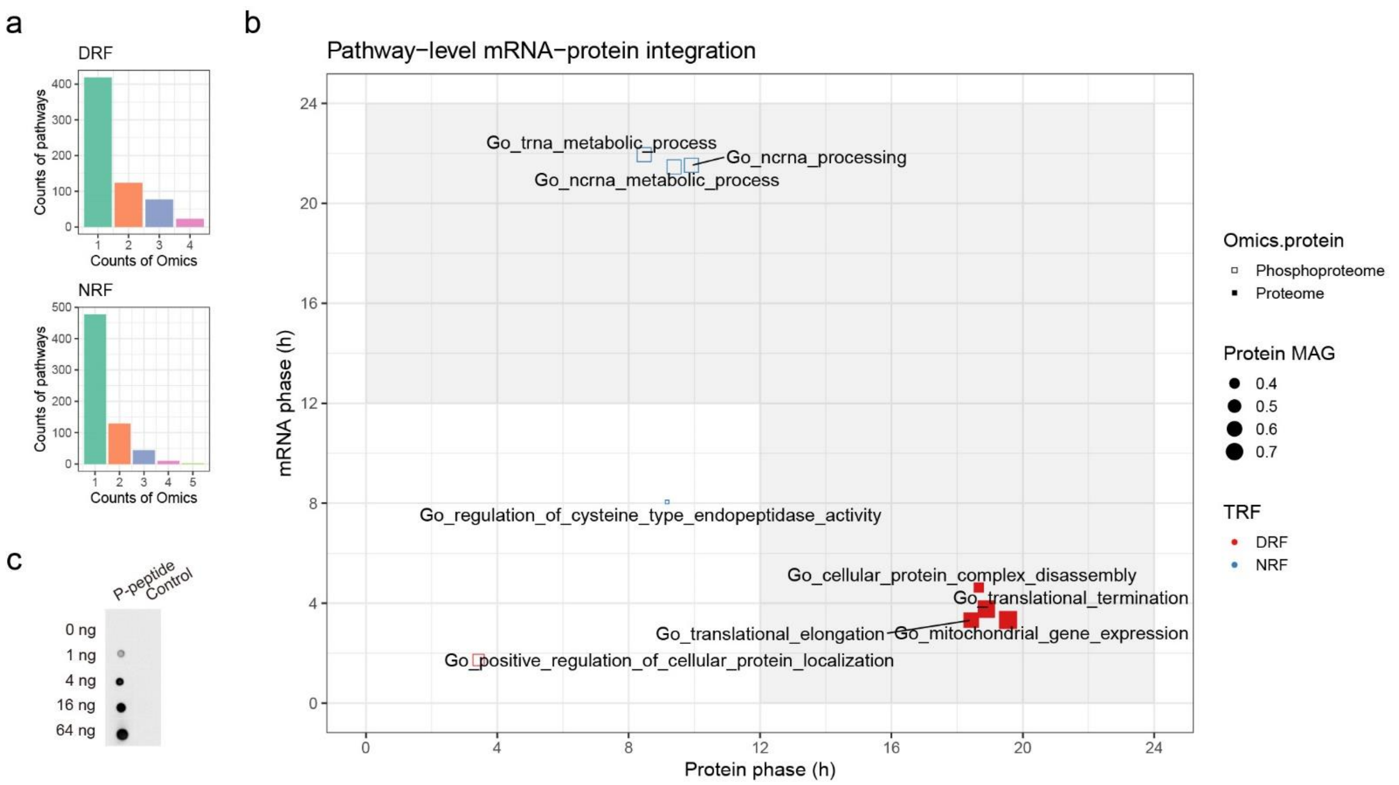

Supplementary Figure 7. Integrative analysis of rhythmic pathways in mouse livers under DRF and NRF. (a) Histograms showing the counts of shared rhythmic pathways under DRF or NRF, as measured by PSEA (Kuiper test, $q < 0.05$). (b) Phase plot showing the phase of rhythmic pathways found in the diurnal transcriptome and diurnal proteomes including different post-translational modifications (PTM), as measured by PSEA (Kuiper test, $q < 0.05$). Protein MAG, magnitude of enrichment in (PTM)-proteomics. (c) Representative dot blots of purified rabbit polyclonal antibody recognizing a PER2-pSer971 peptide (P-peptide). The experiment was repeated twice with similar results. Source data are provided as a Source Data file.

Supplementary Methods

Targeted lipidomics

Lipids were extracted from approximately 20 mg tissues using a modified version of the Bligh and Dyer's method[1]. Briefly, tissues were homogenized in 750 μL of chloroform: methanol 1:2 (v/v) with 10% deionized water on a bead ruptor (OMNI, USA). The homogenate was then incubated at 1500 rpm for 1 h at 4 °C. At the end of the incubation, 350 μL of deionized water and 250 μL of chloroform were added to induce phase separation. The samples were then centrifuged and the lower organic phase containing lipids was extracted into a clean tube. Lipid extraction was repeated once by adding 500 μL of chloroform to the remaining tissues in aqueous phase, and the lipid extracts were pooled into a single tube and dried in the SpeedVac under OH mode. Samples were stored at -80 °C until further analysis.

Polar lipids were analyzed using an Exion UPLC system coupled with a triple quadrupole/ion trap mass spectrometer (6500 Plus Qtrap; SCIEX)[2,3]. Separation of individual lipid classes of polar lipids by normal phase (NP)-HPLC was carried out using a Phenomenex Luna 3 μm-silica column (internal diameter 150 × 2.0 mm) with the following conditions: mobile phase A (chloroform: methanol: ammonium hydroxide, 89.5:10:0.5) and mobile phase B (chloroform: methanol: ammonium hydroxide: water, 55:39:0.5:5.5). MRM transitions were set up for comparative analysis of various polar lipids.

Individual lipid species were quantified by referencing to spiked internal standards. PC-14:0/14:0, PE-14:0/14:0, PS34:1-d31, PA-17:0/17:0, PG-14:0/14:0, Cer d18:1/15:0-d7, SM d18:1/12:0 and GluCer d18:1/8:0 were obtained from Avanti Polar Lipids. Dioctanoyl phosphatidylinositol (PI) (16:0-PI) was obtained from Echelon Biosciences, Inc. Glycerol lipids including diacylglycerols (DAGs) and triacylglycerols (TAGs) were quantified using a modified version of reverse phase HPLC/MRM. Separation of neutral lipids were achieved on a Phenomenex Kinetex-C18 2.6 μm column (i.d. 4.6x100 mm) using an isocratic mobile phase containing chloroform:methanol:0.1 M ammonium acetate 100:100:4 (v/v/v) at a flow rate of 170 μL for 17 min. Levels of short-, medium-, and long-chain TAGs were calculated by referencing to spiked internal standards of TAG(14:0)3-d5, TAG(16:0)3-d5 and TAG(18:0)3-d5 obtained from CDN isotopes, respectively. DAGs were quantified using d5-DAG17:0/17:0 and d5-DAG18:1/18:1 as internal standard (Avanti Polar Lipids). Free cholesterols and cholesteryl esters were analyzed as described previously with d6-cholesterol and d6-C18:0 cholesteryl ester (CE) (CDN isotopes) as internal standards. Free fatty acids were quantitated using d31-16:0 (Sigma-Aldrich) and d8-20:4 (Cayman Chemicals) as internal standards, while d3-16:0-acylcarnitine (Cayman Chemicals) were used for quantitation of acyl-carnitines.

N-acylethanolamines (NAEs) and N-acyl phosphatidylethanolamines (NAPEs) were extracted from liver tissues using a modified method of Bligh and Dyer's extraction[4]. The organic phase was extracted and dried in SpeedVac under organic mode. Samples were resuspended in acetonitrile: isopropanol (1:2 v/v) containing appropriate concentrations of internal standards including d7-PE33:1 from Avanti Polar Lipids, d8-20:4-EA, d4-16:0-EA, d4-22:6-EA and d5-MAG-20:4 from Cayman Chemicals. Samples were analyzed on a ThermoFisher U3000 DGLC coupled to Sciex 6500 Plus QTRAP under the electrospray ionization mode. Individual lipids were separated on an Agilent Zorbax Eclipse Plus column (100 x 2.1 mm, 1.8 μm) using Mobile Phase A (10 mM ammonium formate: acetonitrile: isopropanol 50:30:20, pH 8) and Mobile Phase B (10 mM ammonium formate: acetonitrile: isopropanol 1:9:90, pH 8). NAEs and NAPEs were quantitated by referencing to spiked internal standards.

Proteasome activity assay

For in vitro proteasome activity assay, liver tissues were homogenized in ice-cold 0.5% NP-40 (in PBS). Supernatants were collected after centrifugation at 4 °C and protein concentration was determined by BCA assay. Proteasome activities (per 30-90 μg

proteins) were detected using a fluorometric proteasome substrate (Succ-Leu-Leu-Val-Tyr-AMC) following the established protocol (Abcam Inc. #ab107921, previously known as Biovision #K245-100). Fluorescence output was detected using a fluorometric plate reader (Ex 350 nm, Em 440 nm). The assay protocol can be accessed via Proteasome Activity Assay Kit (ab107921) | Abcam.

Supplementary References

1. Lam, S. M. *et al.* A multi-omics investigation of the composition and function of extracellular vesicles along the temporal trajectory of COVID-19. *Nat. Metab.* **3**, 909–922 (2021).
2. Lu, J. *et al.* High-Coverage Targeted Lipidomics Reveals Novel Serum Lipid Predictors and Lipid Pathway Dysregulation Antecedent to Type 2 Diabetes Onset in Normoglycemic Chinese Adults. *Diabetes Care* **42**, 2117–2126 (2019).
3. Song, J. W. *et al.* Omics-Driven Systems Interrogation of Metabolic Dysregulation in COVID-19 Pathogenesis. *Cell Metab.* **32**, 188-202.e5 (2020).
4. Lam, S. M. *et al.* Quantitative Lipidomics and Spatial MS-Imaging Uncovered Neurological and Systemic Lipid Metabolic Pathways Underlying Troglomorphic Adaptations in Cave-Dwelling Fish. *Mol. Biol. Evol.* **39**, 1–18 (2022).
